# (*E*)-3-(2-Hy­droxy-5-methyl­phenyl­imino)­indolin-2-one

**DOI:** 10.1107/S1600536811034015

**Published:** 2011-08-27

**Authors:** Peng-Fei Zhang, Cai-Feng Bi, Qiang Wang, Jian Zuo, Nan Zhang

**Affiliations:** a Key Laboratory of Marine Chemistry, Theory and Technology, Ministry of Education, College of Chemistry and Chemical Engineering, Ocean University of China, Qingdao, Shandong 266100, People’s Republic of China

## Abstract

In the title compound, C_15_H_12_N_2_O_2_, the dihedral angle between the two benzene rings is 83.55 (11)° In the crystal, the molecules are linked by O—H⋯O and N—H⋯O hydrogen bonds.

## Related literature

For general background on Schiff base ligands, see: Guo *et al.* (2011 ▶); Drozdzak *et al.* (2005 ▶); Weber *et al.* (2007 ▶); Liu *et al.* (2010 ▶). For standard bond lengths, see: Allen *et al.* (1987 ▶)
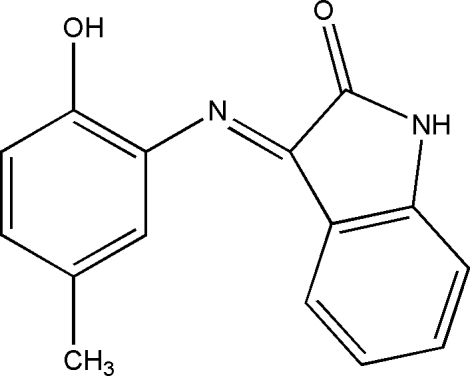


## Experimental

### 

#### Crystal data

C_15_H_12_N_2_O_2_
*M* *_r_* = 252.27
Monoclinic, 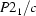

*a* = 12.6211 (11) Å
*b* = 8.7100 (7) Å
*c* = 11.2835 (10) Å
β = 90.780 (1)°
*V* = 1240.28 (18) Å^3^
*Z* = 4
Mo *K*α radiation
μ = 0.09 mm^−1^
*T* = 298 K
0.50 × 0.47 × 0.17 mm

#### Data collection

CCD area-detector diffractometer
Absorption correction: multi-scan (*SADABS*; Sheldrick, 2003 ▶) *T*_min_ = 0.956, *T*_max_ = 0.985
5982 measured reflections
2182 independent reflections
1449 reflections with *I* > 2σ(*I*)
*R*_int_ = 0.038

#### Refinement

*R*[*F* ^2^ > 2σ(*F* ^2^)] = 0.039
*wR*(*F* ^2^) = 0.114
*S* = 1.02
2182 reflections
173 parameters
H-atom parameters constrained
Δρ_max_ = 0.16 e Å^−3^
Δρ_min_ = −0.16 e Å^−3^

### 

Data collection: *SMART* (Siemens, 1996 ▶); cell refinement: *SMART*; data reduction: *SAINT* (Siemens, 1996 ▶); program(s) used to solve structure: *SHELXS97* (Sheldrick, 2008 ▶); program(s) used to refine structure: *SHELXL97* (Sheldrick, 2008 ▶); molecular graphics: *SHELXTL* (Sheldrick, 2008 ▶); software used to prepare material for publication: *SHELXTL*.

## Supplementary Material

Crystal structure: contains datablock(s) I, global. DOI: 10.1107/S1600536811034015/ru2008sup1.cif

Structure factors: contains datablock(s) I. DOI: 10.1107/S1600536811034015/ru2008Isup2.hkl

Supplementary material file. DOI: 10.1107/S1600536811034015/ru2008Isup3.cml

Additional supplementary materials:  crystallographic information; 3D view; checkCIF report

## Figures and Tables

**Table 1:** Hydrogen-bond geometry (Å, °)

*D*—H⋯*A*	*D*—H	H⋯*A*	*D*⋯*A*	*D*—H⋯*A*
O2—H2⋯O1^i^	0.82	1.86	2.6729 (19)	169
N1—H1⋯O2^ii^	0.86	2.06	2.842 (2)	151

Symmetry codes: (i) 
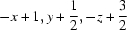
; (ii) 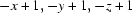.

**Fig. 1.**
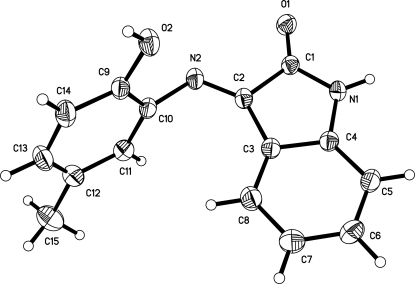
The structure of the title compound, showing 30% probability displacement ellipsoids and the atom-numbering scheme.

**Fig. 2.**
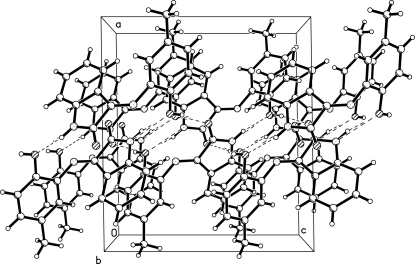
A view of the crystal structure showing chain to the c linked via O—H···O and N—H···O contacts.

## supplementary crystallographic information

### Comment

Being one of the most prevalent sustems in coordination chemistry, Schiff base ligands have received much attention in recent years (Guo *et al.*, 2011), primarily due to their importance in metal complexes with catalytic activities (Drozdzak *et al.*, 2005), special magnetism (Weber *et al.*, 2007) and biological properties, for example, anticancer activities (Liu *et al.*, 2010). In the present paper, the synthesis and structure of a new Schiff base ligand is reported.

The crystal structure of the new ligand is given in Fig.1. The molecular structure of the compound is not coplanar, the dihedral angle between the two benzene rings is 83.55 °, it is almost perpendicular. The bond lengths and angles (Table 1) are within normal values (Allen *et al.*,1987). In the crystal structure, the adjacent molecules are linked through 0—H···0 and N—H···O hydrogen bonding (Table 2), to generate one-dimensional chain in direction (Fig.2).

### Experimental

2,3-indolinedione (5 mmol, 0.736 g) was dissolved in anhydrous ethanol (20 ml), then an anhydrous ethanol solution (10 ml) of 2-amino-4-methylphenol (5 mmol, 0.612 g) was slowly added. The mixture was refluxed for 4 h at 333k,and then cooled down to room temperature. A dark brown solid separated out. The solid was filtered off, washed several times with anhydrous ethanol and dried in vacuum drier. The dark brown single-crystal of the title ligand suitable for X-ray diffraction was trained in anhydrous ethanol at room temperature.

### Refinement

All H-atoms were positioned geometrically and refined using a riding model, with C—H = 0.96 Å (methyl), 0.93 Å (methenyl), 0.93 Å (aromatic), and *U*_iso_(H) =1.2*U*_eq_(C).

### Crystal data

**Table:**

C_15_H_12_N_2_O_2_	*F*(000) = 528
*M**_r_* = 252.27	*D*_x_ = 1.351 Mg m^−^^3^
Monoclinic, *P*2_1_/*c*	Mo *K*α radiation, λ = 0.71073 Å
Hall symbol: -P2ybc	Cell parameters from 1807 reflections
*a* = 12.6211 (11) Å	θ = 2.8–25.8°
*b* = 8.7100 (7) Å	µ = 0.09 mm^−^^1^
*c* = 11.2835 (10) Å	*T* = 298 K
β = 90.780 (1)°	Block, dark-brown
*V* = 1240.28 (18) Å^3^	0.50 × 0.47 × 0.17 mm
*Z* = 4	

### Data collection

**Table:**

CCD area-detector diffractometer	2182 independent reflections
Radiation source: fine-focus sealed tube	1449 reflections with *I* > 2σ(*I*)
graphite	*R*_int_ = 0.038
φ and ω scans	θ_max_ = 25.0°, θ_min_ = 2.8°
Absorption correction: multi-scan (*SADABS*; Sheldrick, 2003)	*h* = −14→14
*T*_min_ = 0.956, *T*_max_ = 0.985	*k* = −10→10
5982 measured reflections	*l* = −10→13

### Refinement

**Table:**

Refinement on *F*^2^	Secondary atom site location: difference Fourier map
Least-squares matrix: full	Hydrogen site location: inferred from neighbouring sites
*R*[*F*^2^ > 2σ(*F*^2^)] = 0.039	H-atom parameters constrained
*wR*(*F*^2^) = 0.114	*w* = 1/[σ^2^(*F*_o_^2^) + (0.0486*P*)^2^ + 0.3363*P*] where *P* = (*F*_o_^2^ + 2*F*_c_^2^)/3
*S* = 1.02	(Δ/σ)_max_ < 0.001
2182 reflections	Δρ_max_ = 0.16 e Å^−^^3^
173 parameters	Δρ_min_ = −0.16 e Å^−^^3^
0 restraints	Extinction correction: *SHELXL*, Fc^*^=kFc[1+0.001xFc^2^λ^3^/sin(2θ)]^-1/4^
Primary atom site location: structure-invariant direct methods	Extinction coefficient: 0.017 (2)

### Special details

**Table:**

Geometry. All e.s.d.'s (except the e.s.d. in the dihedral angle between two l.s. planes) are estimated using the full covariance matrix. The cell e.s.d.'s are taken into account individually in the estimation of e.s.d.'s in distances, angles and torsion angles; correlations between e.s.d.'s in cell parameters are only used when they are defined by crystal symmetry. An approximate (isotropic) treatment of cell e.s.d.'s is used for estimating e.s.d.'s involving l.s. planes.
Refinement. Refinement of *F*^2^ against ALL reflections. The weighted *R*-factor *wR* and goodness of fit *S* are based on *F*^2^, conventional *R*-factors *R* are based on *F*, with *F* set to zero for negative *F*^2^. The threshold expression of *F*^2^ > σ(*F*^2^) is used only for calculating *R*-factors(gt) *etc*. and is not relevant to the choice of reflections for refinement. *R*-factors based on *F*^2^ are statistically about twice as large as those based on *F*, and *R*- factors based on ALL data will be even larger.

### Fractional atomic coordinates and isotropic or equivalent isotropic displacement parameters (Å^2^)

**Table:**

	*x*	*y*	*z*	*U*_iso_*/*U*_eq_	
N1	0.53216 (12)	0.37862 (19)	0.37637 (13)	0.0391 (4)	
H1	0.4865	0.3785	0.3191	0.047*	
N2	0.62133 (12)	0.28888 (19)	0.66041 (14)	0.0404 (4)	
O1	0.45391 (11)	0.19850 (16)	0.49452 (12)	0.0474 (4)	
O2	0.58728 (11)	0.51425 (16)	0.82237 (12)	0.0490 (4)	
H2	0.5830	0.5750	0.8776	0.073*	
C1	0.52386 (15)	0.2918 (2)	0.47439 (16)	0.0358 (5)	
C2	0.61738 (14)	0.3381 (2)	0.55449 (16)	0.0355 (5)	
C3	0.67930 (15)	0.4476 (2)	0.48520 (17)	0.0384 (5)	
C4	0.62382 (14)	0.4693 (2)	0.37841 (17)	0.0369 (5)	
C5	0.65726 (16)	0.5680 (2)	0.29187 (18)	0.0465 (5)	
H5	0.6188	0.5816	0.2218	0.056*	
C6	0.75054 (18)	0.6460 (3)	0.3136 (2)	0.0564 (6)	
H6	0.7756	0.7137	0.2567	0.068*	
C7	0.80770 (18)	0.6262 (3)	0.4179 (2)	0.0611 (7)	
H7	0.8704	0.6805	0.4299	0.073*	
C8	0.77297 (16)	0.5266 (3)	0.5045 (2)	0.0519 (6)	
H8	0.8118	0.5131	0.5744	0.062*	
C9	0.68333 (15)	0.4423 (2)	0.82729 (17)	0.0400 (5)	
C10	0.70454 (15)	0.3352 (2)	0.73941 (16)	0.0398 (5)	
C11	0.80213 (16)	0.2627 (3)	0.73843 (19)	0.0496 (6)	
H11	0.8161	0.1920	0.6789	0.059*	
C12	0.87940 (17)	0.2925 (3)	0.8235 (2)	0.0566 (6)	
C13	0.85588 (18)	0.3980 (3)	0.9110 (2)	0.0596 (7)	
H13	0.9064	0.4195	0.9695	0.072*	
C14	0.75915 (17)	0.4720 (3)	0.91347 (19)	0.0516 (6)	
H14	0.7451	0.5421	0.9734	0.062*	
C15	0.98450 (19)	0.2096 (4)	0.8213 (3)	0.0907 (10)	
H15A	1.0391	0.2757	0.8522	0.136*	
H15B	1.0006	0.1817	0.7412	0.136*	
H15C	0.9806	0.1186	0.8691	0.136*	

### Atomic displacement parameters (Å^2^)

**Table:**

	*U*^11^	*U*^22^	*U*^33^	*U*^12^	*U*^13^	*U*^23^
N1	0.0391 (9)	0.0482 (10)	0.0300 (9)	0.0008 (8)	−0.0053 (7)	0.0017 (8)
N2	0.0432 (9)	0.0452 (10)	0.0326 (9)	0.0010 (8)	−0.0054 (7)	−0.0002 (8)
O1	0.0487 (8)	0.0530 (9)	0.0403 (8)	−0.0093 (7)	−0.0018 (6)	0.0006 (7)
O2	0.0542 (9)	0.0530 (9)	0.0395 (8)	0.0082 (7)	−0.0098 (7)	−0.0066 (7)
C1	0.0389 (11)	0.0384 (12)	0.0300 (11)	0.0044 (9)	0.0008 (8)	−0.0030 (9)
C2	0.0397 (10)	0.0358 (11)	0.0311 (11)	0.0069 (9)	−0.0014 (8)	−0.0030 (9)
C3	0.0399 (11)	0.0400 (12)	0.0352 (11)	0.0019 (9)	−0.0009 (9)	0.0006 (9)
C4	0.0391 (11)	0.0383 (12)	0.0335 (11)	0.0057 (9)	0.0028 (8)	−0.0010 (9)
C5	0.0520 (13)	0.0494 (13)	0.0380 (12)	0.0034 (11)	0.0011 (10)	0.0066 (10)
C6	0.0611 (14)	0.0521 (15)	0.0562 (15)	−0.0070 (12)	0.0071 (12)	0.0141 (12)
C7	0.0560 (14)	0.0610 (16)	0.0660 (16)	−0.0177 (12)	−0.0049 (12)	0.0104 (13)
C8	0.0507 (13)	0.0544 (15)	0.0502 (13)	−0.0066 (11)	−0.0090 (10)	0.0062 (11)
C9	0.0425 (12)	0.0432 (12)	0.0341 (11)	−0.0041 (9)	−0.0055 (9)	0.0061 (10)
C10	0.0424 (11)	0.0466 (13)	0.0302 (11)	−0.0049 (10)	−0.0037 (8)	0.0053 (9)
C11	0.0457 (12)	0.0609 (15)	0.0421 (13)	0.0020 (11)	0.0026 (10)	0.0035 (11)
C12	0.0414 (12)	0.0746 (17)	0.0537 (14)	−0.0034 (12)	−0.0054 (10)	0.0126 (13)
C13	0.0507 (14)	0.0753 (18)	0.0523 (15)	−0.0169 (13)	−0.0190 (11)	0.0100 (14)
C14	0.0597 (14)	0.0541 (14)	0.0405 (13)	−0.0083 (12)	−0.0115 (10)	0.0005 (11)
C15	0.0469 (14)	0.133 (3)	0.092 (2)	0.0122 (17)	−0.0062 (14)	0.013 (2)

### Geometric parameters (Å, °)

**Table:**

N1—C1	1.345 (2)	C7—C8	1.383 (3)
N1—C4	1.401 (2)	C7—H7	0.9300
N1—H1	0.8600	C8—H8	0.9300
N2—C2	1.270 (2)	C9—C14	1.380 (3)
N2—C10	1.427 (2)	C9—C10	1.390 (3)
O1—C1	1.223 (2)	C10—C11	1.384 (3)
O2—C9	1.365 (2)	C11—C12	1.383 (3)
O2—H2	0.8200	C11—H11	0.9300
C1—C2	1.531 (3)	C12—C13	1.384 (3)
C2—C3	1.466 (3)	C12—C15	1.511 (3)
C3—C8	1.383 (3)	C13—C14	1.381 (3)
C3—C4	1.398 (3)	C13—H13	0.9300
C4—C5	1.372 (3)	C14—H14	0.9300
C5—C6	1.379 (3)	C15—H15A	0.9600
C5—H5	0.9300	C15—H15B	0.9600
C6—C7	1.383 (3)	C15—H15C	0.9600
C6—H6	0.9300		
			
C1—N1—C4	112.15 (16)	C3—C8—H8	120.7
C1—N1—H1	123.9	C7—C8—H8	120.7
C4—N1—H1	123.9	O2—C9—C14	123.33 (19)
C2—N2—C10	120.77 (17)	O2—C9—C10	117.30 (17)
C9—O2—H2	109.5	C14—C9—C10	119.36 (19)
O1—C1—N1	126.19 (18)	C11—C10—C9	119.46 (18)
O1—C1—C2	128.17 (17)	C11—C10—N2	120.89 (19)
N1—C1—C2	105.61 (16)	C9—C10—N2	119.23 (17)
N2—C2—C3	135.01 (18)	C12—C11—C10	121.9 (2)
N2—C2—C1	119.12 (17)	C12—C11—H11	119.1
C3—C2—C1	105.61 (15)	C10—C11—H11	119.1
C8—C3—C4	119.07 (19)	C11—C12—C13	117.6 (2)
C8—C3—C2	134.48 (18)	C11—C12—C15	120.7 (2)
C4—C3—C2	106.45 (16)	C13—C12—C15	121.7 (2)
C5—C4—C3	122.87 (19)	C14—C13—C12	121.5 (2)
C5—C4—N1	127.13 (18)	C14—C13—H13	119.2
C3—C4—N1	109.99 (17)	C12—C13—H13	119.2
C4—C5—C6	116.9 (2)	C9—C14—C13	120.2 (2)
C4—C5—H5	121.5	C9—C14—H14	119.9
C6—C5—H5	121.5	C13—C14—H14	119.9
C5—C6—C7	121.6 (2)	C12—C15—H15A	109.5
C5—C6—H6	119.2	C12—C15—H15B	109.5
C7—C6—H6	119.2	H15A—C15—H15B	109.5
C6—C7—C8	120.9 (2)	C12—C15—H15C	109.5
C6—C7—H7	119.6	H15A—C15—H15C	109.5
C8—C7—H7	119.6	H15B—C15—H15C	109.5
C3—C8—C7	118.6 (2)		
			
C4—N1—C1—O1	−177.50 (18)	C4—C5—C6—C7	−0.2 (3)
C4—N1—C1—C2	4.1 (2)	C5—C6—C7—C8	0.0 (4)
C10—N2—C2—C3	−4.0 (3)	C4—C3—C8—C7	0.9 (3)
C10—N2—C2—C1	−177.06 (16)	C2—C3—C8—C7	−178.5 (2)
O1—C1—C2—N2	−7.8 (3)	C6—C7—C8—C3	−0.4 (4)
N1—C1—C2—N2	170.58 (17)	O2—C9—C10—C11	177.98 (17)
O1—C1—C2—C3	177.25 (18)	C14—C9—C10—C11	−1.3 (3)
N1—C1—C2—C3	−4.36 (19)	O2—C9—C10—N2	−9.3 (3)
N2—C2—C3—C8	8.8 (4)	C14—C9—C10—N2	171.34 (18)
C1—C2—C3—C8	−177.5 (2)	C2—N2—C10—C11	−82.8 (2)
N2—C2—C3—C4	−170.7 (2)	C2—N2—C10—C9	104.6 (2)
C1—C2—C3—C4	3.1 (2)	C9—C10—C11—C12	0.7 (3)
C8—C3—C4—C5	−1.1 (3)	N2—C10—C11—C12	−171.90 (19)
C2—C3—C4—C5	178.45 (18)	C10—C11—C12—C13	0.2 (3)
C8—C3—C4—N1	179.66 (17)	C10—C11—C12—C15	179.0 (2)
C2—C3—C4—N1	−0.8 (2)	C11—C12—C13—C14	−0.4 (3)
C1—N1—C4—C5	178.56 (19)	C15—C12—C13—C14	−179.1 (2)
C1—N1—C4—C3	−2.2 (2)	O2—C9—C14—C13	−178.12 (19)
C3—C4—C5—C6	0.7 (3)	C10—C9—C14—C13	1.2 (3)
N1—C4—C5—C6	179.83 (19)	C12—C13—C14—C9	−0.3 (3)

### Hydrogen-bond geometry (Å, °)

**Table:**

*D*—H···*A*	*D*—H	H···*A*	*D*···*A*	*D*—H···*A*
O2—H2···O1^i^	0.82	1.86	2.6729 (19)	169.
N1—H1···O2^ii^	0.86	2.06	2.842 (2)	151.

Symmetry codes: (i) −*x*+1, *y*+1/2, −*z*+3/2; (ii) −*x*+1, −*y*+1, −*z*+1.
